# Acute paretic syndrome in juvenile White Leghorn chickens resembles late stages of acute inflammatory demyelinating polyneuropathies in humans

**DOI:** 10.1186/1742-2094-7-7

**Published:** 2010-01-28

**Authors:** Sophie R Bader, Sonja Kothlow, Sascha Trapp, Susanne CN Schwarz, Hans-Christian Philipp, Steffen Weigend, Ahmad R Sharifi, Rudolf Preisinger, Wolfgang Schmahl, Bernd Kaspers, Kaspar Matiasek

**Affiliations:** 1 Chair of General Pathology & Neuropathology, Institute of Veterinary Pathology, Ludwig-Maximilians-University, Munich, Germany; 2 Institute of Veterinary Physiology, Ludwig-Maximilians-University, Munich, Germany; 3 Institute for Medical Microbiology, Infectious and Epidemic Diseases, Ludwig-Maximilians-University, Munich, Germany; 4 Lohmann Tierzucht, Cuxhaven, Germany; 5 Institute of Farm Animal Genetics, Friedrich-Loeffler-Institute, Neustadt, Germany; 6 Institute of Animal Breeding and Genetics, Georg-August-University, Göttingen, Germany

## Abstract

**Background:**

Sudden limb paresis is a common problem in White Leghorn flocks, affecting about 1% of the chicken population before achievement of sexual maturity. Previously, a similar clinical syndrome has been reported as being caused by inflammatory demyelination of peripheral nerve fibres. Here, we investigated in detail the immunopathology of this paretic syndrome and its possible resemblance to human neuropathies.

**Methods:**

Neurologically affected chickens and control animals from one single flock underwent clinical and neuropathological examination. Peripheral nervous system (PNS) alterations were characterised using standard morphological techniques, including nerve fibre teasing and transmission electron microscopy. Infiltrating cells were phenotyped immunohistologically and quantified by flow cytometry. The cytokine expression pattern was assessed by quantitative real-time PCR (qRT-PCR). These investigations were accomplished by MHC genotyping and a PCR screen for Marek's disease virus (MDV).

**Results:**

Spontaneous paresis of White Leghorns is caused by cell-mediated, inflammatory demyelination affecting multiple cranial and spinal nerves and nerve roots with a proximodistal tapering. Clinical manifestation coincides with the employment of humoral immune mechanisms, enrolling plasma cell recruitment, deposition of myelin-bound IgG and antibody-dependent macrophageal myelin-stripping. Disease development was significantly linked to a 539 bp microsatellite in MHC locus LEI0258. An aetiological role for MDV was excluded.

**Conclusions:**

The paretic phase of avian inflammatory demyelinating polyradiculoneuritis immunobiologically resembles the late-acute disease stages of human acute inflammatory demyelinating polyneuropathy, and is characterised by a Th1-to-Th2 shift.

## Background

With an incidence of about 1.5 per 100.000 citizen, Guillain-Barré syndrome (GBS) is the most common cause of acute flaccid paralysis in the western hemisphere and probably worldwide [1]. Amongst different GBS subtypes, acute inflammatory demyelinating polyneuropathy (AIDP) is the most prevalent form in Europe, North America and Australia. AIDP is histopathologically characterised by the combination of primary demyelination and infiltration by lymphocytes and macrophages [2]. Chronic inflammatory demyelinating polyneuropathy (CIDP) is pathologically and epidemiologically [3] similar to AIDP but it shows a protracted or relapsing disease course [4], and is usually responsive to immunosuppression by glucocorticoid treatment [5].

Both GBS and CIDP are immune-mediated disorders involving humoral and cellular effector mechanisms [2]. Thereby, both cascades appear to follow a stage-specific sequence. After exposure to a causative environmental (or endogenous) antigen, autoimmune mechanisms firstly are activated in a T-helper cell 1 (Th1)-specific manner [6]. Even though, in clinical settings, the initial trigger usually remains unknown, certain specific infections and vaccinations have been found to precede episodes of GBS, and it has been hypothesized that the associated immunogens cross-react with epitopes of peripheral myelin by means of a molecular mimicry [2,5,7,8]. Recent studies have revealed that during the plateau or recovery period of late stages of GBS there is a shift from Th1 towards T-helper cell 2 (Th2)-guided events, which suggests that the myelin-specific, Th2-mediated humoral response might ameliorate the disease course [9,10].

To date, most insights into the immunobiology of inflammatory demyelinating neuropathies (IDP) have been gained from experimental animal studies. The most frequently employed model for GBS is experimental autoimmune neuritis (EAN) generated in Lewis rats. These animals are immunized with peripheral myelin or with the purified myelin proteins P0, P2 and/or PMP22. Alternatively, EAN can be induced by adoptive transfer of activated P2-specific neuritogenic T-lymphocytes [11]. Various different EAN subtypes mirror the different types and stages of natural IDP. Active EAN and "adoptive-transfer EAN", for example, reflect the Th1-dominated stages of GBS [12,13] whereas immunisation with a single large dose of PNS myelin or galactocerebrosides in complete Freund's adjuvants lead to a chronic progressive or relapsing disease course, compatible with human CIDP [14]. None of the experimental models, however, is appropriate in all regards as they may include central nervous system (CNS) involvement, which is not typical of natural IDP [12]. Moreover they involve well-defined immunogenic triggers that are more likely to be targets of secondary exposure than the disease-causing immunogen in natural IDP.

Hence, a spontaneous animal model would be useful to gain deeper insights into the complex immunological aspects of disease development, if it were to prove reproducible and broadly available for translational research.

To date, spontaneous forms of CIDP have been described in dogs and cats [15], but the apparently low prevalence in these species precludes in-depth research. Other types of polyradiculoneuritis, like coonhound paralysis, are comparable to the axonal but not the demyelinating form of the Guillain-Barré syndrome [15].

Being alerted by own observations and previous reports on a sporadic paretic syndrome in up to 4% of young White Leghorn layer chickens [16], we addressed the question whether this disorder might resemble mammalian IDP, and performed detailed investigations on the disease phenotype, genetic background and exposure to relevant infective agents.

We demonstrate here that the avian neuropathic disease bears striking similarities to late stage of human AIDP. Even though the primary immunologic trigger has not been identified, we identified an MHC-linked genetic factor, rendering the animals susceptible to this avian inflammatory demyelinating polyradiculoneuropathy (AvIDP).

## Methods

### Animal selection

The present investigation enrolled 40 female White Leghorn chickens that originated from a commercial hybrid flock comprising 5000 individuals. The chickens were sorted into AvIDP-affected and disease-free individuals, following neurological examination [17]. All animals were euthanized using a lethal dose of intravenous sodium pentobarbitone (2 ml/kg BM) [18].

### Histopathology and transmission electron microscopy

#### Histotechnical processing

The cranial vaults and cranial nerve emergences were carefully dissected and the brain and cranial nerve roots were inspected in situ. Thereafter, the trigeminal and oculomotor nerve roots were harvested for further processing.

The brains were gently removed and immersed in 10% neutral-buffered formalin for 48 hours. Fixed samples were trimmed in transverse sections at the following landmarks: (1) the caudal border of the olfactory bulbs, (2) the optic chiasm, (3) the emergence of the oculomotor nerve from the midbrain, (4) the cerebellar peduncles, and (5) the obex.

The entire spinal cord was approached through laminectomy. The spinal nerve roots and associated dorsal root ganglia (DRG) were freed from overlying soft tissue and inspected in situ. Samples of the spinal cord, nerve roots and DRG of three consecutive segments of the widest part of the cervical (C13, C14, Th1) and lumbosacral (S5, S6, S7) intumescence were taken for further investigations.

Morphological examination of the above mentioned cranial and spinal nerves, the brachial plexus, sciatic nerve and abdominal branch of vagus nerve included paraffin and semithin histology, teased fibre evaluation and transmission electron microscopy (TEM).

For routine histology, brain, spinal cord, DRG and peripheral nerves underwent fixation in 10% neutral-buffered formalin, processing in an automatic tissue processor, embedding in paraffin (Paraplast^®^, Tyco Healthcare Group LP, Mannsfield, U.S.A.), sectioning at 5 μm (PNS) and 8 μm (CNS), mounting on triethoxysilane-coated slides (Sigma-Aldrich^®^, Steinheim, Germany) and staining with haematoxylin & eosin (HE).

Further samples of spinal nerve roots and DRG as well as the oculomotor nerve (CNIII), trigeminal nerve (CNVm/o), brachial plexus, sciatic and vagus nerve were cut into 1- to 2-cm segments and immersed in 2.5% glutaraldehyde in 0.1 M Soerensen's phosphate buffer (ph 7.4) for 1 hour. After fixation, the samples were rinsed with Soerensen's phosphate buffer containing 0.2 M buffered D(+)-saccharose. The spinal nerves were cut into 10-mm pieces for nerve fibre teasing and blocks of 2 mm length were harvested from all nerve and DRG probes for epoxy embedding.

Teasing samples were postfixed in 2% osmium tetroxide for 1 hour and, after repeated buffer rinses, placed into 100% water-free glycerol for 24 hours. After that, a total number of 50 single-nerve fibres and/or small fibre clusters were teased apart under a stereo magnifying glass (Olympus SZH, Zeiss Stemi DV4, Germany).

Evidence of increased endoneurial round cells and Schwann cell hyperplasia was further verified by toluidine blue staining and subsequent teasing in aqueous medium.

The 2-mm pieces were postfixed in 1% osmium tetroxide for 2 hours and underwent repeated buffer rinses and a graded alcohol series before being embedded in epoxy resin (Glycidether 100, Serva^®^, Heidelberg, Germany). Semithin sections (0.5 μm) were mounted on microscope slides and stained with azur II methylenblue and safranin O.

Moreover, ultrathin sections (80 nm) were mounted on copper rings covered by Formvar^® ^foils (Plano, Wetzlar, Germany) and contrasted with lead-citrate and uranyl-acetate.

#### Morphological examination

Light microscopical examination was performed on a Zeiss Axiophot^® ^(Zeiss Instruments, Oberkochen, Germany) by two separate investigators (SB, KM) blinded for the origin of these samples. HE-stained paraffin sections were inspected for interstitial and vascular changes as well as for abnormalities affecting myelinated fibres and fibre groups. The histological assessment was accomplished using semithin sections that allowed a deeper insight into single fibre pathology through better preservation of the myelin substance, and served as scout samples for subsequent electron microscopy. Evaluation criteria were in accordance to established protocols for peripheral nerve examination [19]. Relevant abnormalities were scored semiquantitatively into: 0 (absent), 1 (mild; < 25% of fibres in a fascicle cross section affected), 2 (moderate; 25-50% of fibres affected), 3 (severe; >50% of fibres affected) as previously described by Maier et al. [20]. In case of inter-rater discrepancy the slides were reviewed and the final results were recorded after an agreement was achieved.

Nerve fibre teasing was performed to assess longitudinal parameters and distribution patterns, to evaluate the state of axons and myelin sheaths at different sub-segmental levels, and to evaluate nodal gaps [21,22].

Ultrastructural analysis was performed and documented photographically with a Zeiss EM 10^® ^(Germany) at 80 kV at magnifications from 2.500× to 80.000×. Thereby, the myelin spirals could be evaluated in detail, as well as the nerve fibre axoplasm and Schwann cell perikarya [23]. Moreover, TEM analysis allowed the evaluation of unmyelinated fibre clusters [24].

### Immune cell phenotyping

#### Immunohistochemistry (IHC)

Fresh samples of spinal nerve roots and DRG, oculomotor nerve, brachial plexus and sciatic nerves were embedded into OCT compound medium (TissueTek^®^; Sakura Finetek, The Netherlands) prior to snap-freezing in liquid nitrogen and storage at -80°C until further processing.

Sections of 12 μm were performed using a cryostat (Shandon Southern Products Ltd., Runcorn, Cheshire, UK) and these were mounted on SuperFrost^®^Plus-slides (Menzel-Gläser, Braunschweig, Germany).

Endogenous peroxidase activity was blocked by immersion in 0.3% hydrogen peroxide in methanol for 20 min before incubation with 10% normal goat serum (1:20) for 30 min. Primary antibodies were directed against: chB6 (AV20) (1:100) [25], CD3 (CT3) (1:5) [26], CD4 (2-6) (1:2) [27], CD8 (3-298) (1:50) [28], TCRγ/δ (TCR1) (1:100) [29], TCRα/Vβ1 (TCR2) (1:100) [30], TCRα/Vβ2 (TCR3) (1:100) [31,32], macrophage (KUL01) (1:50) [33], MHCII (2G11) (1:500), IgG (G1) (1:300) and IgM (M1) (1:500) [34]. All antibodies were purified from cell culture supernatants. The antibodies were applied to the slides overnight, at 4°C, followed by a 30-min incubation with biotinylated secondary antibody (1:200; biotinylated goat-anti-mouse IgG, Vector Laboratories, Burlingame, CA, USA). ABC Kit (Vectastain^®^, Vector Laboratories, CA, USA; 30 min) and HistoGreen (Linaris Biologische Produkte GmbH, Germany) were used as detection kits. Sections were then counterstained with nuclear fast red, dehydrated and mounted with Histofluid^® ^(Marienfeld, Germany). The density of respective immunopositive cells was assessed semiquantitatively using a modified protocol of Pavlakis et al. [35]: mild (1+): < 2% of the cells positive; moderate (2+): 2-10% of the cells positive; severe (3+): > 10% of the cells positive. Localisation of the immune cells was evaluated in regard to infiltrated compartments and distribution patterns.

Furthermore, the biotinylated lectin *Ricinus communis *agglutinin-1 (RCA 120, 1:3000; Vector Laboratories, CA, USA) was used as a specific marker for microglial cells within the spinal cord [36]. Eight-micrometer-thick sections of paraffin-embedded samples of the spinal cord of three consecutive segments of the widest part of the cervical (C13, C14, Th1) and lumbosacral (S5, S6, S7) intumescence were deparaffinised and hydrated through xylene and graded alcohol series. IHC was performed as recommended by the manufacturer.

#### Flow cytometry

Spinal nerve roots and DRG from eight affected birds were subjected to cell typing using flow cytometry. Spinal nerve roots, including DRG, were cut into small pieces with scalpel and tweezers. Tissue samples were digested with collagenase type IV (Sigma, Poole, UK) (0,05% in Hank's balanced salt solution (HBSS) with Ca^2+ ^and Mg^2+ ^and 5% FCS) in a shaking water bath at 37°C for 30 min, followed by homogenisation with a 1-ml syringe. After sedimentation of larger fragments on ice for 10 min, the supernatant was transferred to a new tube and cells were washed twice in PBS. Finally the cells were resuspended in PBS with 1% BSA and 0.01% NaN_3_.

Two-colour immunofluorescence staining of the cells was performed according to standard procedures [37].

At first, cells were stained with a monoclonal antibody (mAb) against CD45 combined with mAbs against chB6 (AV20) [25,38], IgM (M1) [34], CD4 (2-6) [27], CD8 (3-298) [28], TCRγ/δ (TCR1) [29], TCRα/Vβ1 (TCR2) [30], TCRα/Vβ2 (TCR3) [31], MHCII (2G11) [39] and/or macrophage (KUL01) [33], followed by staining with an isotype-specific anti-mouse-IgG2a-FITC conjugate and an anti-mouse-IgG1 or IgG2b-phycoerythrin (PE) -conjugate (Southern Biotechnology Associates, Birmingham, Alabama, USA), respectively. Analysis was performed with a FACScan (Becton Dickinson, Heidelberg, Germany) and FlowJo software (Tree Star Inc, Oregon, USA).

### Fluoroscence microscopy

To demonstrate the spatial relationships between infiltrating cells and nerve fibres, unfixed samples of the sciatic nerve underwent whole-mount immunofluorescence investigations. The fascicles were grossly separated from epi- and endoneurium and gently teased apart under a stereo magnifying glass. They were mounted with normal goat serum (1:20) for 30 min followed by an incubation with rabbit anti-laminin antibody (1:200; Abcam, Cambridge, UK) overnight, at 4°C, and with fluorescein-5-isothiocyanat (FITC)-conjugated goat anti-rabbit antiserum (1:400, 30 min; Sigma, Poole, UK), thereafter. Finally, diamidino-2-phenylindol (1:50; DAPI BioChemica, AppliChem GmbH, Darmstadt, Germany) was applied on the sample for 30 min.

The nerve fibre bundles were then teased in aqueous medium and examined with a scanning microscope Zeiss LSM^® ^510 equipped with a Zeiss Axiovert 100 M^®^, a 488 nm argon laser and a 770 nm biphotonic laser. Settings were at 490 nm (track 1/FITC) and 365 nm (track 2/DAPI) wave-length.

Additionally to IHC, cryosections of sciatic nerve and spinal nerve roots, including DRG, were tested for presence of IgM and IgG with immunofluorescence. The slides were mounted with normal goat serum (1:20) for 30 min followed by an incubation with the particular primary antibody (mouse anti-IgM1 [1:500] or mouse anti-IgG1 [1:300]) for 2 h at room temperature. Thereafter, R-phycoerythrin (RPE)-conjugated goat anti-mouse antiserum (1:400; Alexa Fluor, Eugene, Oregon, USA) and, at last, DAPI (1:50) were applied to the sample for 30 min.

Examination took place using the scanning microscope Zeiss LSM^® ^510 (see above) at wavelength settings of 495 nm (track 1/RPE) and 365 nm (track 2/DAPI).

### Quantitative real-time RT-PCR (qRT-PCR)

Pieces of spinal nerve roots, including DRG, were extracted, immediately snap frozen in liquid nitrogen, and stored at -80°C until further processing. For RNA isolation, TRIzol™ Reagent^® ^(Invitrogen, Karlsruhe, Germany) was used according to the manufacturer's protocol. The quantity of extracted RNA was determined by photometry, while the RNA quality was analysed using a 2100 Bioanalyzer^® ^(Agilent Technologies, Böblingen, Germany). Only RNA samples with an RNA integrity number (RIN) exceeding 7.0 were used for qRT-PCR analysis.

Genomic DNA elimination and reverse transcription were performed using a QuantiTect Reverse Transcription Kit^® ^(Qiagen, Hilden, Germany) according to the manufacturer's instructions. The reverse transcription reaction was carried out for 30 min at 42°C and terminated by incubating the samples for 3 min at 95°C.

Primers for qRT-PCR (Table 1) were designed using PrimerExpress software^® ^(Applied Biosystems, Warrington, UK) and were obtained from MWG-Biotech^® ^(Ebersberg, Germany).

**Table 1:** Primers used for RT-qPCR analysis.

Gene	Forward	Reverse
18S rRNA	TGTCTAAGTACACACGGGCGGTACA	GGCGCTCGTCGGCATGTATTA

CD3ε	CATCGCTGCGGATCTTCTCA	GCTGCATCTTCTGGGCTCGT

chB6	GATCGCCTGCCCTCCAAT	TGGCTTTCCACGTCAGCTATC

Blimp-1	GTGGTATTGCCGAGACTTTGC	GGGTTTGTGTGAGGTTCATCATT

AID	CGTCTGAAACCCAGCAAGAGT	TGTCCATGTCAGCTGGGTTCT

iNOS	AAGCAAACGGCCAAGATCCA	CCCACCTCAAGGAGCATGTTG

IFNγ	TGGCGTGAAGAAGGTGAAAGA	TCTGAGACTGGCTCCTTTTCCT

IL-1	CTGAGTCATGCATCGTTTATGTTTC	AAATACCTCCACCCCGACAAG

IL-6	GCTTCGACGAGGAGAAATGC	GCCAGGTGCTTTGTGCTGTA

IL-10	CGGGAGCTGAGGGTGAAGT	CAGCCAAAGGTCCCCTTAAAC

IL-13	GCCACAGTGCTGGACAACAT	ATGCCGTGCAGGCTCTTC

Quantitative RT-PCR was performed using a 7300 Real-Time PCR System^® ^(Applied Biosystems, Warrington, UK) with SYBR-Green as a double-stranded DNA-specific fluorescent dye. Amplification mixes contained 1 μL cDNA, 12.5 μL QuantiTect SYBR Green PCR Kit^® ^(Qiagen, Hilden, Germany), 1.5 μL forward and reverse primer (final concentration 300 nM), and 8.5 μL water. The detector system was programmed to start with an activation step for 15 min at 95°C followed by PCR program with 40 cycles of DNA denaturation (15 sec at 94°C), primer annealing (30 sec at 59°C), and elongation (30 sec at 72°C). Each qRT-PCR was run in triplicate. Specificity of the resulting qRT-PCR products was verified by melting curve analysis.

To normalize the data, the cycle threshold (CT) values of the housekeeping gene 18S rRNA were subtracted from the target gene CT value of the sample (= dCT).

### Genotyping

For MHC genotyping, commercial White Leghorn layer hybrids were used, all originating from the same variety of commercial hybrids. The first set of samples was collected from clinically affected chickens at an age of six weeks. Furthermore, a sampling from healthy animals was performed. In total, samples from 152 healthy and 113 affected chickens were collected.

The microsatellite marker LEI0258 is known to be located within the MHC, between the BG and BF regions, and an association between LEI0258 alleles and serologically defined MHC haplotype has been reported [40]. The aim of the genotyping was to evaluate a possible association between MHC and the occurrence of the paralytic syndrome as previously observed in peripheral neuropathy [16].

A drop of blood was obtained from the wing vein of each bird and absorbed onto Whatman FTA^® ^filter cards (Whatman International Ltd., Maidstone, UK), and dried and stored in an aluminium foil envelope at room temperature until analysis. DNA isolation was carried out using the phenol-chloroform method [41].

PCR was performed using HotStarTaq Master Mix Kit (Qiagen, Hilden, Germany) on an Eppendorf Mastercycler using 20 ng DNA. To amplify locus LEI0258 the forward primer sequence was 5'cacgcagcagaacttggtaagg 3', while the reverse primer sequence was 5'agctgtgctcagtcctcagtgc 3'. An initial denaturation was performed at 95°C for 15 min, followed by 35 cycles at 94°C for 1 min, 58°C for 1 min and 72°C for 1 min. The PCR finished with a final extension at 72°C for 10 min. DNA fragments were analyzed by electrophoresis on a 4% agarose gel for 3 h at 6 V/cm, and visualized with ethidium bromide staining on UV light. Resulting fragments corresponded to alleles of 261 bp, 357 bp, and 539 bp, respectively.

### Statistical analyses

The data were analyzed by a generalized linear model using proc LOGISTIC (SAS Institute, 2008) with genotype and farm as main effect. An Exact Conditional Test was performed for including variables in the model. Only genotype remained as a significant variable in the final model, using a significance level of p < 0.05. Odds ratios and 95% confidence intervals were calculated for different levels of significant main effects using the pairwise CONTRAST statement. Wald statistics were used in determining the statistical significance of estimated odds ratios. The raw frequencies of genotypes were counted using Proc FREQ (SAS, SAS Institute, Heidelberg, Germany).

### PCR analysis for MDV strain discrimination

Samples of spleen, sciatic nerve, and spinal nerve roots, including DRG, were collected from 10 AvIDP-affected and non-affected White Leghorn chickens, all of which were vaccinated against Marek's disease with avirulent MDV-CVI988/Rispens (AviPro MD RISPENS RL, Lohmann Animal Health, Cuxhaven, Germany). Similarly, organ samples were collected from 10 six-week-old specific-pathogen-free (SPF) control chickens (Lohmann VALO SPF). Genomic DNA from 10 (spleen tissue) to 20 mg (nerve tissue) of each organ sample was isolated using the QIAamp DNA Mini Kit (Qiagen, Hilden, Germany) according to the manufacturer's protocol. DNA concentrations were measured using a NanoDrop spectrophotometer (NanoDrop Technologies, Wilmington, Delaware, USA) and adjusted to a final concentration of 100 ng/μl. One hundred ng of DNA of each sample were then analysed by conventional PCR (40 cycles of 30 sec at 94°C, 30 sec at 55°C, and 45 sec at 72°C) using the Thermoprime Plus Mastermix (ABgene, Surrey, UK) and the oligonucleotide primers given in Table 2. Primers UL49amp_fw and UL49amp_rv were designed to amplify a region of 618 base pairs (bp) in the UL49-ORF of MDV. Primers UL49disc_fw and UL49disc_rv span a fragment of 320 bp located within the 618 bp region and were conceived to discriminate CVI988/Rispens strains from virulent MDV field strains through a DNA polymorphism found in the UL49-ORF of CVI988/Rispens [42]. Controls for the PCR analysis included MDV-DNA isolated from the very virulent MDV strain RB-1B- and from virulent MDV strain BC-1-transformed lymphoblastoid cell lines 54-O [Trapp et al., unpublished data] and MSB-1 [43], as well as CVI988/Rispens-DNA isolated from two different commercially available vaccine preparations (AviPro MD RISPENS RL, Lohmann Animal Health, Cuxhaven, Germany, and Nobilis Rismavac, Intervet International GmBH, Unterschleissheim Germany).

**Table 2:** Oligonucleotide primers used for Marek's disease virus PCR analysis

UL49amp_fw	5'-ATGGCGACGCGAAGTTG-3'
UL49amp_rv	5'-CCATCAACACGTACTCAGCGA-3'

UL49disc_fw	5'-CTTGTACGTTCAGATTTGG**TTG**-3'

UL49disc_rv	5'-TCATCAGCATCTAGCACTTGG-3'

Letters in bold indicate three nucleotides at the 3'-end of UL49disc_fw that mismatch with the UL49-sequence of MDV-CVI988/Rispens (...ACT-3').

## Results

### Clinical presentation

The 20 affected animals revealed histories of impaired limb movements with an onset prior to achievement of sexual maturity at an age between 5 and 13 weeks. All affected animals uniformly showed a progressive asymmetric paresis of one or multiple extremities on neurological examination. Wing and leg involvement was characterized by weakness progressing to flaccid paresis. In the due course of the disease the proximal limbs show intermittent relative extensor hypertonicity. In addition, limb weakness was accompanied by fasciculations of limb and neck musculature. Postural reactions in the affected legs were absent or markedly decreased.

The 20 age-matched control chickens behaved normally during open-field observation and did not exhibit any dysfunctions on clinical and neurological examination.

### Morphological findings

**Figure 1:**
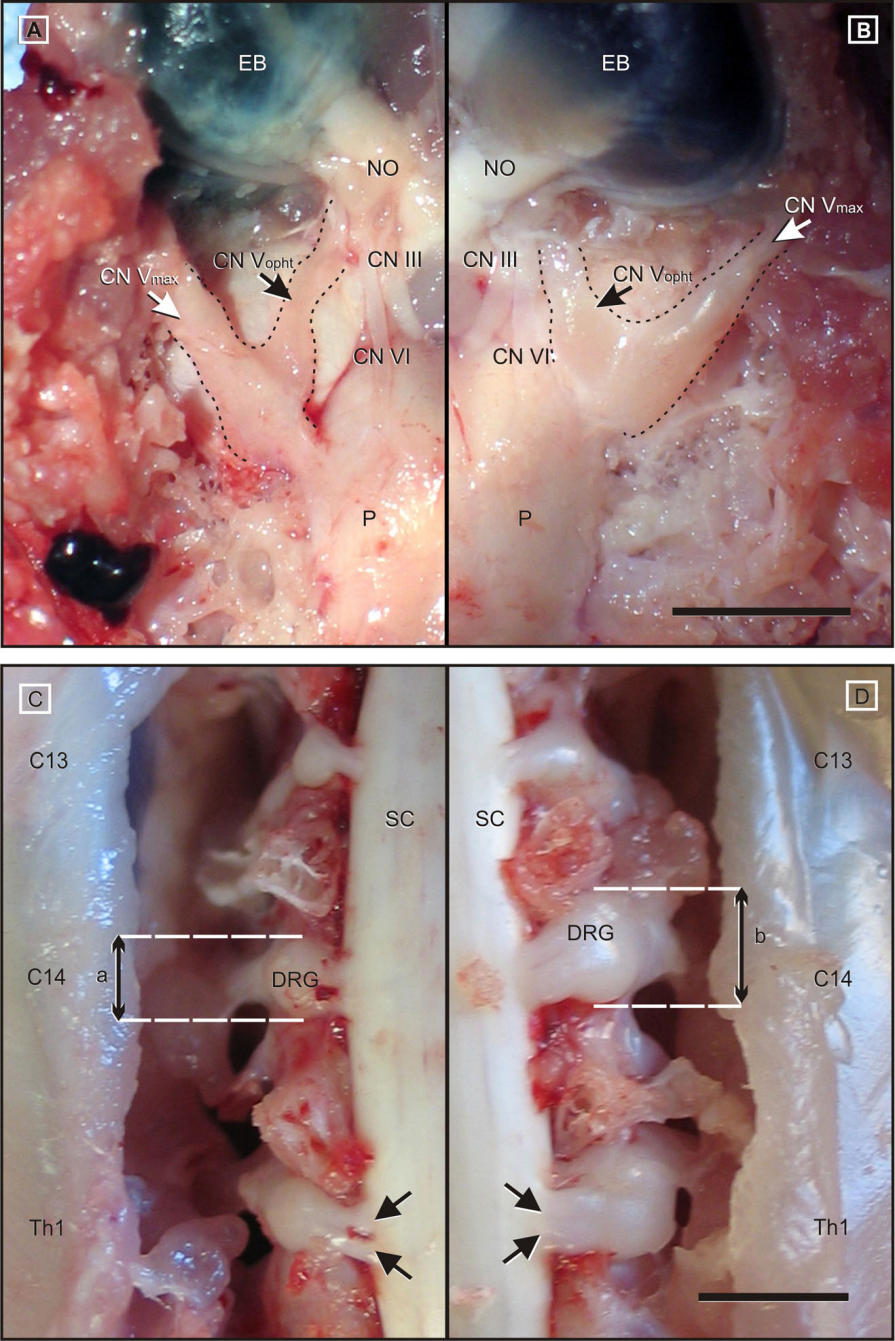
**Gross findings in AvIDP-affected chickens**. Compared to controls (A, C) a marked thickening of craniospinal nerve roots was recognized in all affected birds (B, D). A, B: Inspection of the ventral brain surface reveals severely enlarged maxillary (CN Vmax) and ophthalmic (CN Vopht) nerve branches. The oculomotor (CN III) and abducens (CN VI) nerve are also thickened to a lesser extend, and show a mild greyish discoloration. C, D: The dorsal view at the cervical intumescence between C13 and Th1 indicates severe enlargement of the dorsal rootlets (arrows) and the associated dorsal root ganglia (DRG). Affected ganglia (b) measure up to fourfold the diameters of unaffected control nerves (a). A, B: EB = eye ball; NO = optic nerve; P = Pons; scale bar = 1.0 cm. C, D: SC: spinal cord; scale bar 0.5 cm.

On necropsy, all clinically ill chickens revealed a severe, bilaterally symmetrical oedematous thickening of the cranial and spinal nerve roots. Amongst cranial nerves, the maxillary and ophthalmic branches of CN V were most severely affected (Figure 1A and 1B). To a slightly lesser degree, the oculomotor (CN III) and facial nerve (CN VII) were involved.

In affected chickens, the spinal nerve roots emerging from the cervical and lumbosacral intumescences showed identical changes. Their diameters measured up to four times the normal calibre (Figure 1C and 1D). Thereby, the diameters tapered off centrifugally so that the nerves presented normal diameters immediately distal to the intervertebral foramen.

**Figure 2:**
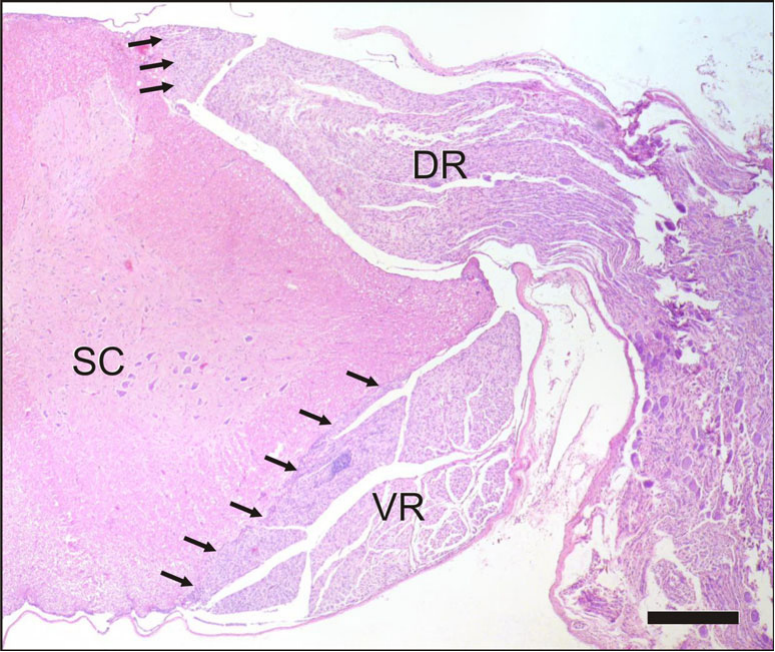
**CNS-PNS transition as the border of inflammation**. The arrows point to the CNS - PNS boundary of the spinal roots. Despite the severe involvement of both dorsal (DR) and ventral (VR) nerve roots, the inflammatory infiltration spares the adjacent central white matter and does not pass the PNS-CNS transition. Scale bar = 0.5 mm.

**Figure 3:**
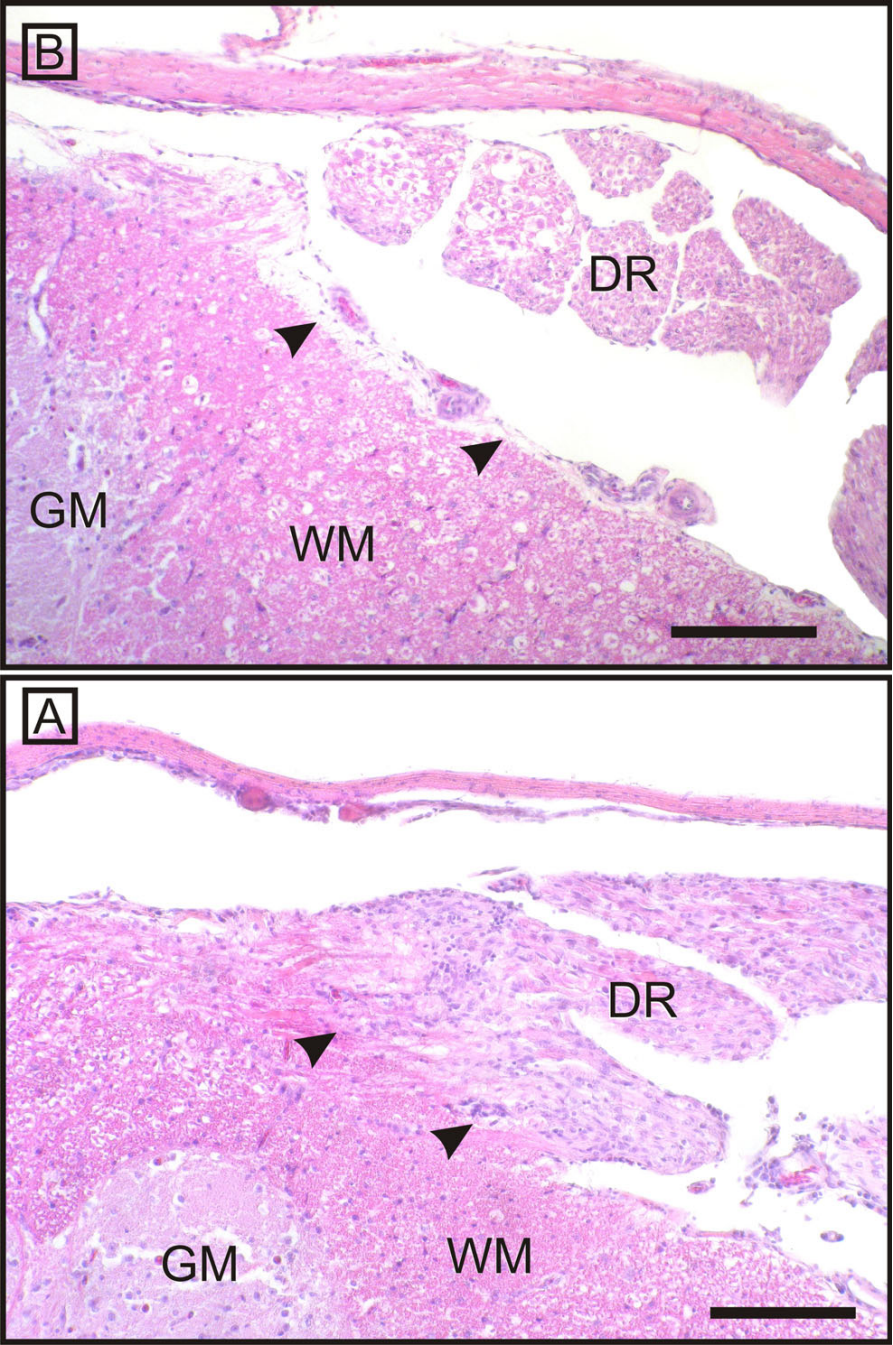
**CNS-PNS transition at the dorsal nerve root as the border of inflammation**. On closer inspection, the dorsal root (DR) of an affected animal (B) shows a mononuclear infiltration in comparison to a control chicken (A). The arrows point to the CNS - PNS boundary. Note that the adjacent white (WM) and grey matters (GM) are not affected. Scale bar = 1 mm.

**Figure 4:**
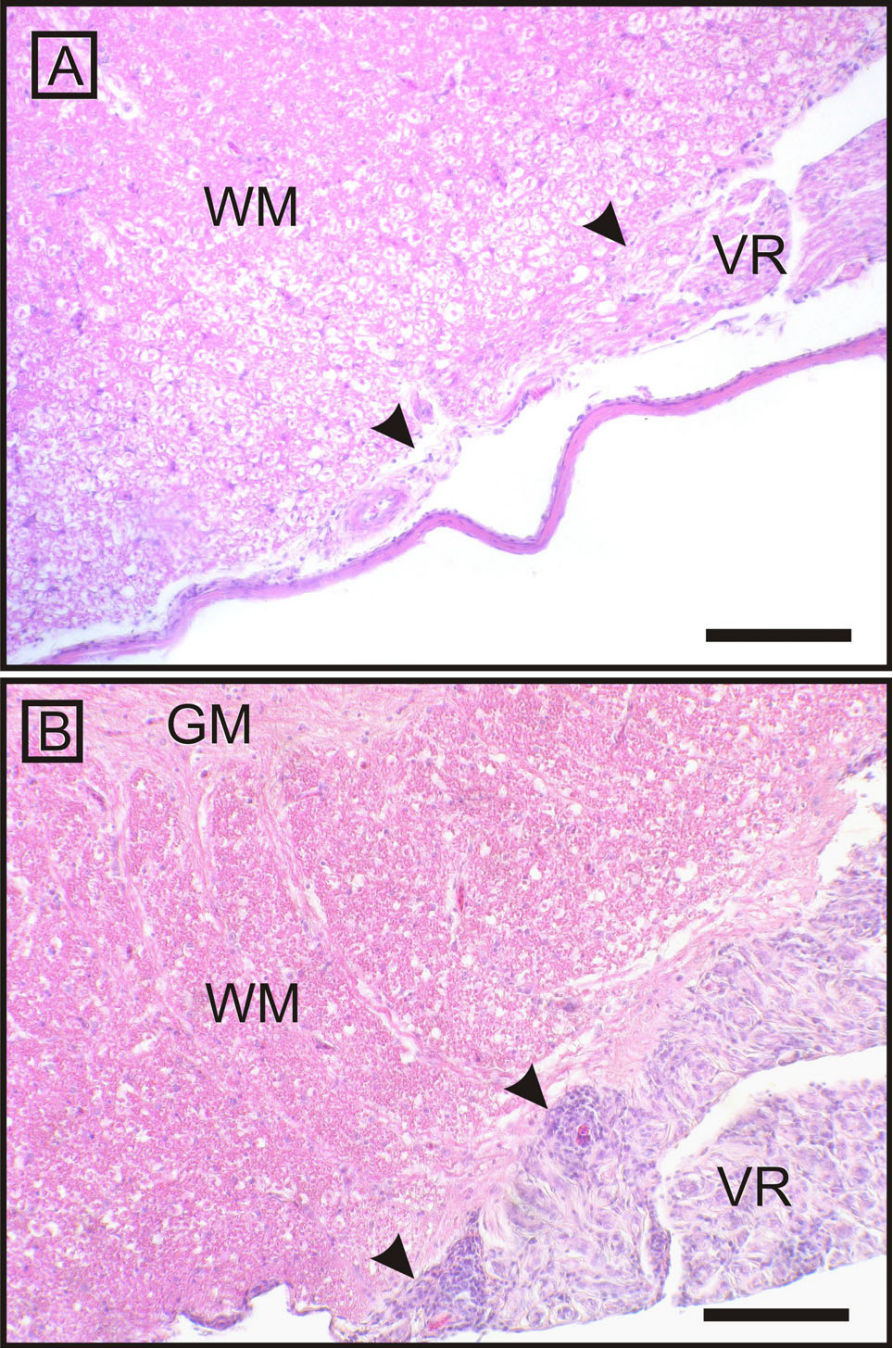
**CNS-PNS transition at the ventral nerve root as the border of inflammation**. The ventral nerve root (VR) of an affected animal (B) presents with a severe mononuclear infiltration and thickening in comparison to a control chicken (A). The arrows point to the CNS - PNS boundary. Again, the inflammatory infiltration spares the adjacent central white (WM) and grey matters (GM) and does not pass the PNS-CNS transition. Scale bar = 1 mm.

**Figure 5:**
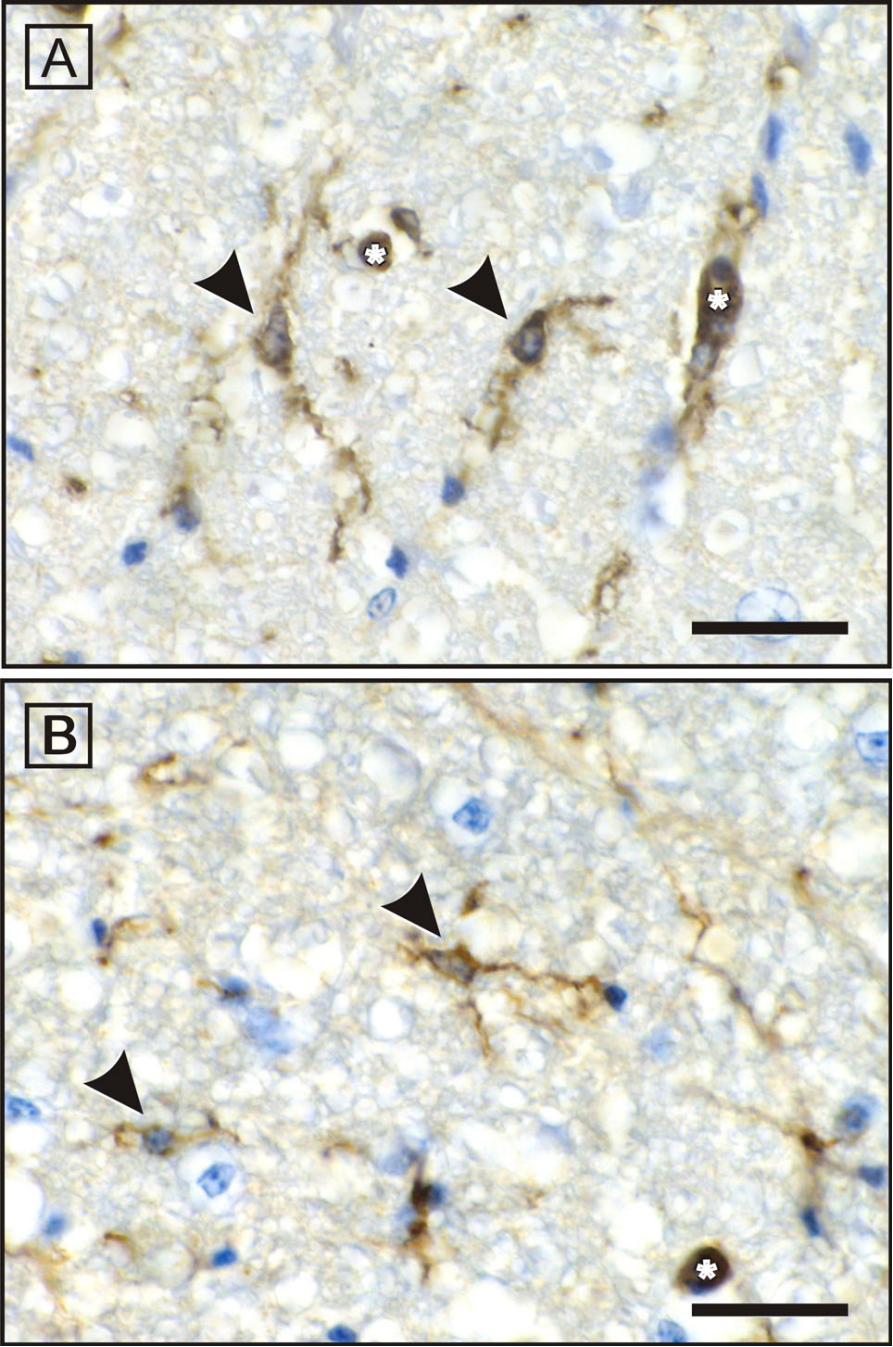
**Immunohistochemical illustration of *Ricinus communis *agglutinin-1 (RCA-1) -positive microglial cells in the white matter of spinal cord**. The RCA-1-positive microglial cells (arrowheads) in both healthy (A) and AvIDP-affected (B) chicken show incospicuous ramified microglial cells. Activated microglial cells or an increased number were not observed. A, B: asterisk = endothelium (also stains RCA-1 positive); scale bar = 250 μm.

Microscopically, all examined parts of the peripheral nervous system of ill animals showed concurrent inflammation and demyelination. Thereby, the former was characterised by multifocal lymphohistiocytic infiltrates, with some perivascular cuffing - particularly frequent and most severe in the ganglia - and scattered plasma cells throughout the endoneurium of the nerves and ganglia. Inflammation seemed to start directly at the CNS-PNS transition, without involving the central neuroparenchyma (Figure 2, 3, 4 and 5), culminated in the roots, and tapered off from proximal to distal (Figure 2, 3 and 4). Hence, the prevertebral and cranial nerve ganglia were amongst the hot spots. Both dorsal and ventral roots revealed similar inflammation scores. Clinically healthy chickens sometimes revealed follicular lymphocytic infiltrates within the dorsal ganglia consistent with findings of other groups [16,44,45].

In affected chickens, at most 50% of the cross sectional area of CNs III and V, the brachial plexus, and the sciatic nerve were pathologically altered, especially in the most proximal parts. Only the vagus nerve was lacking length-dependent features and presented with a diffuse inflammation of the entire endoneurium in all samples. Notably, the infiltration foci colocalised with large clusters of nude and hypomyelinated axons. Teased preparations of affected nerves revealed a multisegmental and, infrequently, also paranodal cell-mediated demyelination involving numerous mononuclear cells with macrophage characteristics that penetrated the Schwann cell basal lamina at the Schmidt-Lanterman incisures. Consistent with ongoing remyelination, inappropriately thin myelinated fibres were recognised in many areas. These remyelinating segments and many demyelinated axons were encircled by supernumerary Schwann cell processes.

**Figure 6:**
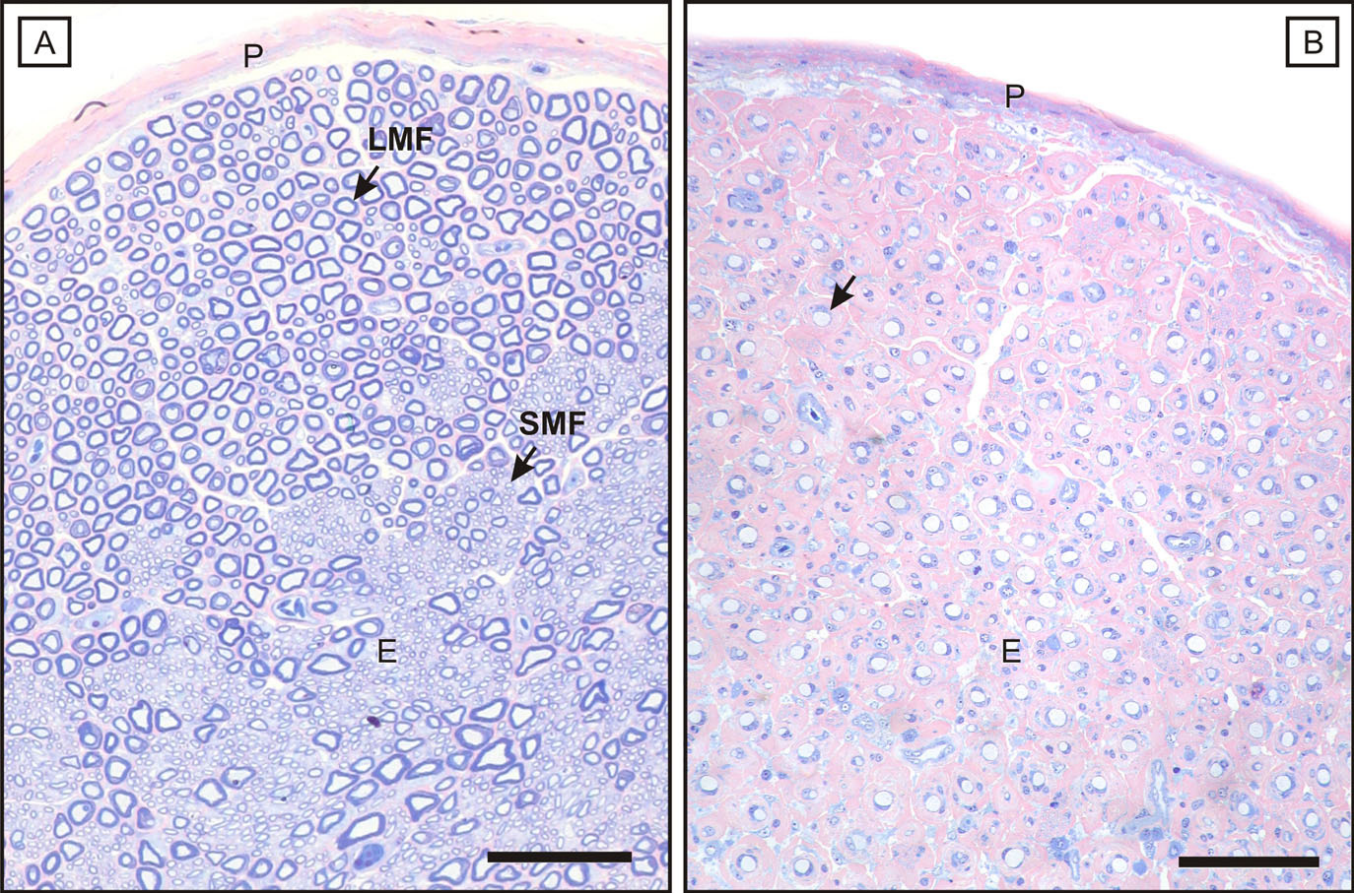
**AvIDP: typical histological PNS findings**. The histological appearance of a healthy mixed fascicular nerve is displayed in figure A. Its endoneurium (E) contains large (LMF) and small (SMF) myelinated fibres that are confined by a peripheral rim of blue (azurophilic) compacted myelin. B: AvIDP leads to a breakdown of the myelin sheath while leaving the axons mainly untouched. Concomitantly with the myelin loss (arrow), an expansion of the eosinophilic extracellular matrix of the endoneurial sheath develops which results in a decrease of nerve fibre density. A, B: P = perineurium; scale bar = 100 μm.

Myelin sheath alterations were accompanied by a significant expansion of the collagenous inner endoneurial sheath (Figure 6). This increase of extracellular matrix compensated for the decreased nerve fibre diameters following myelin loss.

**Figure 7:**
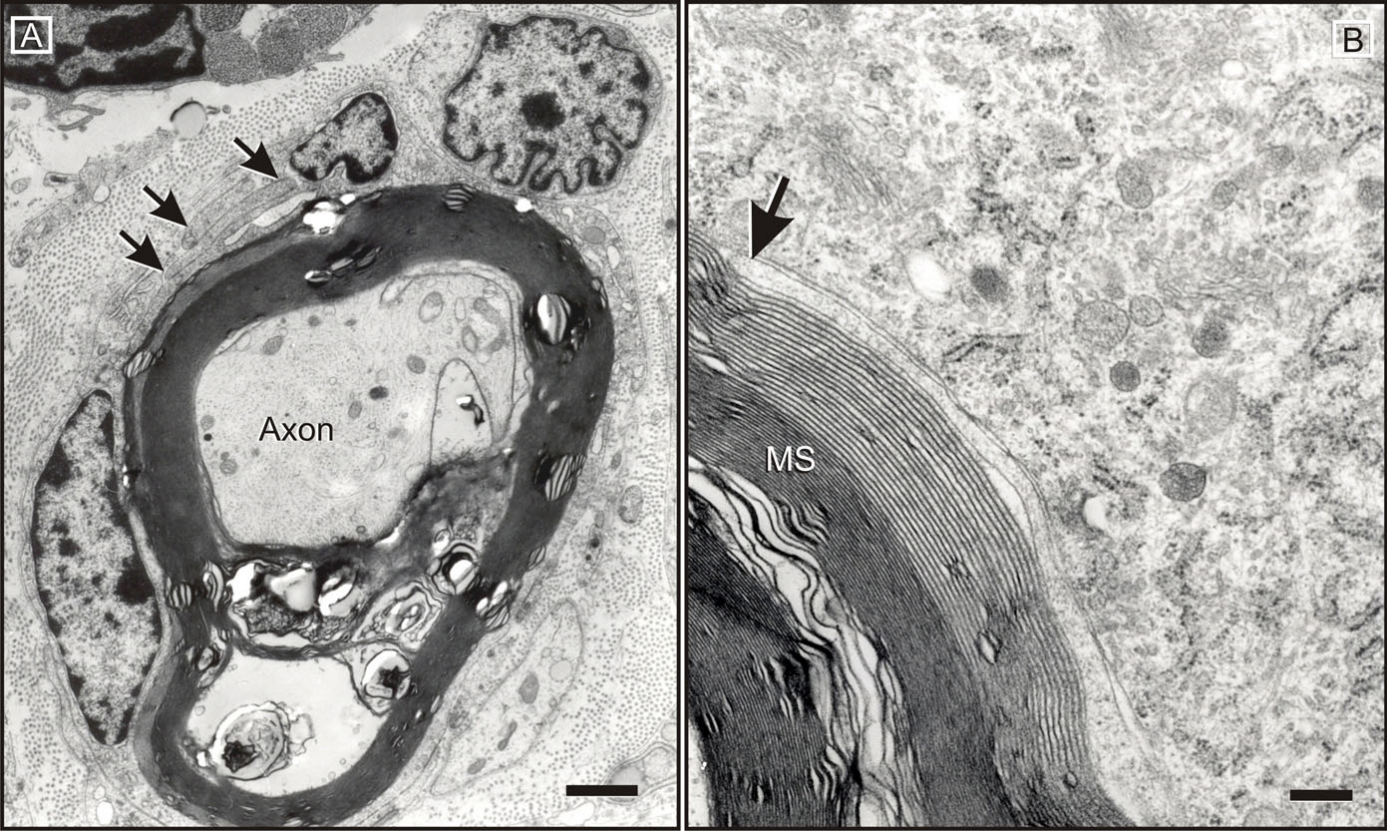
**Macrophage-mediated myelin stripping**. A: Upon invasion of the Schwann cell tubes, macrophage processes (arrows) split the outer mesaxon in order to gain entry to the myelin spiral. B: They invade the myelin following the intraperiod line (arrow). Widening propagates centripetally towards the inner mesaxon. B: MS = myelin sheath; Scale bar = 2 μm.

**Figure 8:**
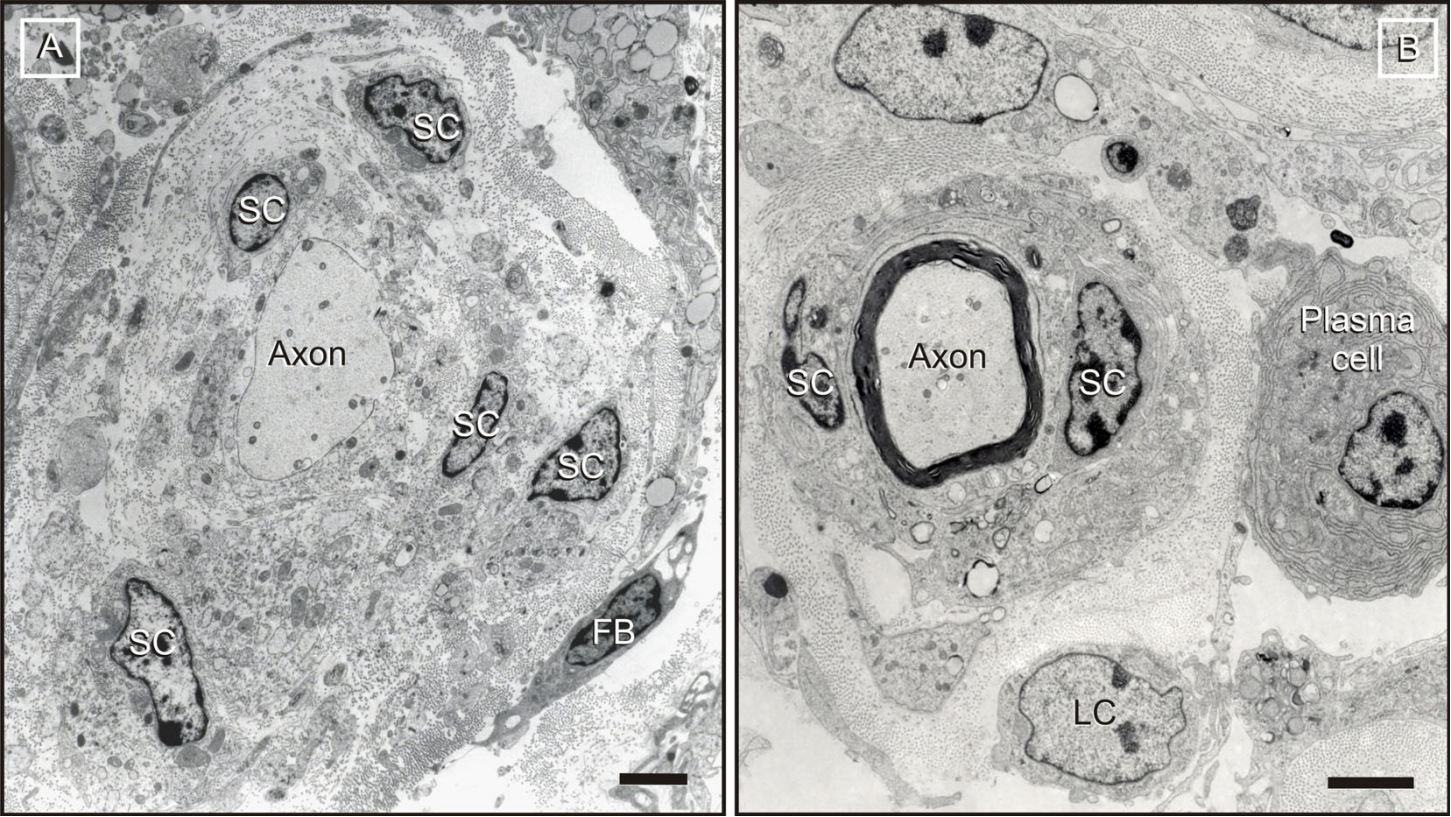
**Ongoing demyelination/remyelination**. A: Demyelination is accompanied by proliferation of Schwann cells (SC), formation of supernumerary processes, and a significant expansion of the collagenous inner endoneurial sheath. Schwann cells show no sign of degeneration. B: Inappropriately thin myelin sheaths and redundant Schwann cells are consistent with ongoing remyelination. Apart from macrophages, the endoneurium of affected nerves shows significant infiltration by plasma cells and lymphocytes (LC). A: FB = fibroblast; Scale bars = 1 μm (A), 250 nm (B).

Transmission electron microscopy of the affected peripheral nerves basically confirmed the light microscopic features. Macrophages multifocally were invading the myelin spiral at the outer mesaxon (Figure 7). Schwann cell nuclei and perikarya showed no signs of degeneration (Figure 8). Apart from a very mild decrease of diameter and slightly more densely packed neurofilaments, the axons retained their normal ultrastructural architecture throughout all affected areas.

Similar findings were not recognised in age-matched control animals.

### Immunostaining

IHC revealed a similar inflammatory cell composition in the samples of spinal nerve roots and associated DRG, CN III, brachial plexus and sciatic nerves. IgM-positive plasma cells were diffusely scattered amongst the endoneurial compartment.

Furthermore, the affected nerves showed a moderate multifocal infiltration of chB6-positive B-cells and a severe multifocal infiltration of CD3-positive T-cells. Overall, the extent of infiltration by the latter appeared about twice as severe as the degree of B-cell involvement.

**Figure 9:**
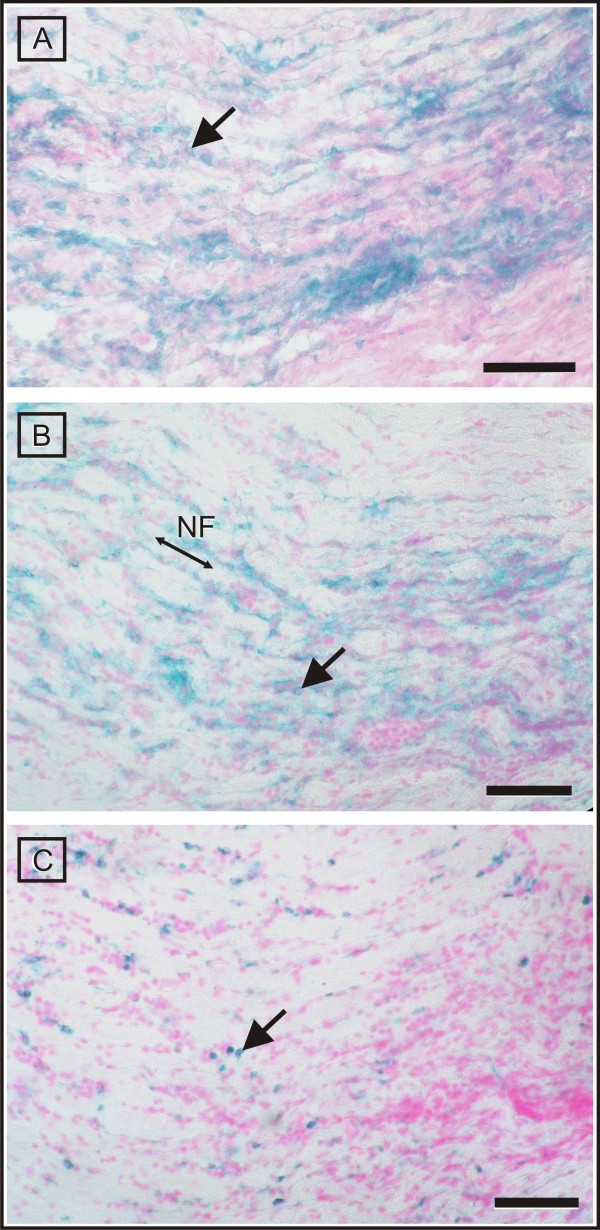
**Immunohistochemical evidence of infiltration by T-cells (CD3^+^), B-cells (chB6^+^) and macrophages (Kul01^+^)**. Immunophenotyping identified the majority of infiltrating lymphocytes as being CD3-positive T-cells (A). They show a characteristic multifocal distribution pattern. With similar spatial characteristics, chB6-positive B-cells comprise the second largest fraction of infiltrating cells (B). Compared to the lymphocytes, the density of Kul01-positive macrophages appears much lower (C). The arrows point to immunopositive cells. All sections were prepared from the same region of the sciatic nerve. The orientation of the nerve fibres (NF) is indicated. Scale bar = 35 μm.

Large perivascular clusters showed an intermingled cell composition of mainly CD3- and chB6-positive cells interspersed with a few KUL01-positive macrophages (Figure 9). All control chickens displayed a few isolated B-cells and T-cells per fascicular cross section surrounding venules, compatible with score of 1+ (data not shown).

Empirically, the T-cell population in affected animals consisted of equal fractions of T-helper cells (CD4^+^) and cytotoxic T-cells (CD8^+^). Most of the T-cells carried αβ T-cell receptor, while a small minority stained positively for TCR1 (γδ T-cells). These T-cell phenotypes intermingled intensely and there was no spatial organisation.

In ill birds, KUL01-positive histiocytes and macrophages were seen frequently in the perineurium and subperineurial spaces, and they intermingled with lymphocytes in large perivascular infiltrates. Compared to control tissue, where we could find single positive cells with the same distribution pattern, the overall density was mildly to moderately increased. The distribution of MHCII-expressing, antigen-presenting cells extended over both macrophage populations and those areas occupied by lymphocytes, showing a severe rise compared to control animals.

**Figure 10:**
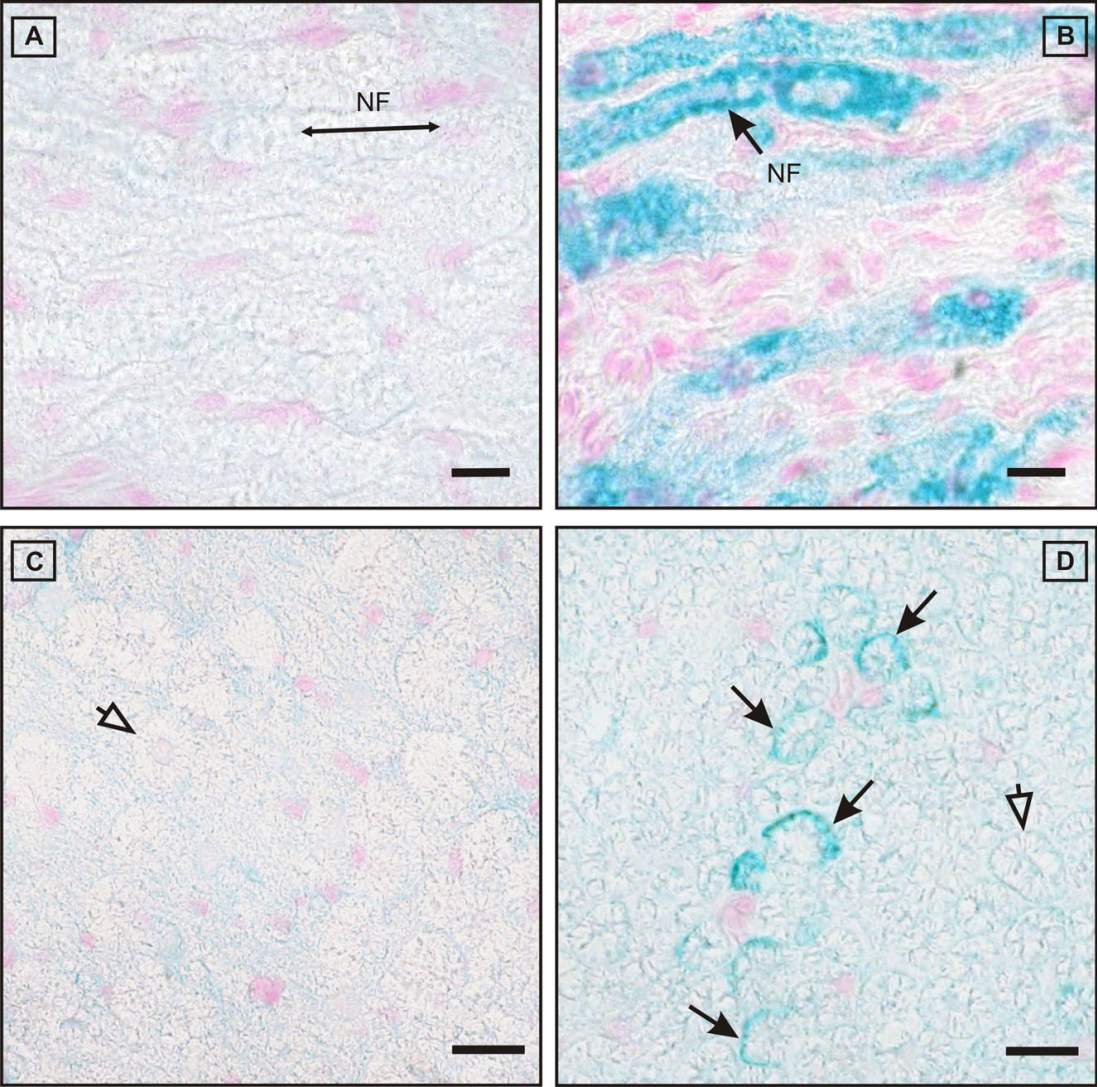
**Deposition of IgG within the myelin sheath of affected fibres**. Longitudinal (A) and cross (C) sections of unaffected samples show immunonegative nerve fibres (NF) throughout (empty arrow), whereas inflamed specimens reveal a significant intramyelinic IgG-deposition (B, D; black arrows) in many fibres within (B) or even outside (D) of significantly infiltrated foci. The longitudinal orientation of the nerve fibers is indicated by the double-headed arrow (A). Scale bars = 10 μm (A, B), 15 μm (C, D).

Apart from cell-associated immunoglobulin staining, patchy IgG-positive signals were confined to the myelin sheaths of large myelinated fibres in spinal nerve roots, including DRG, and peripheral nerve segments (Figure 10).

**Figure 11:**
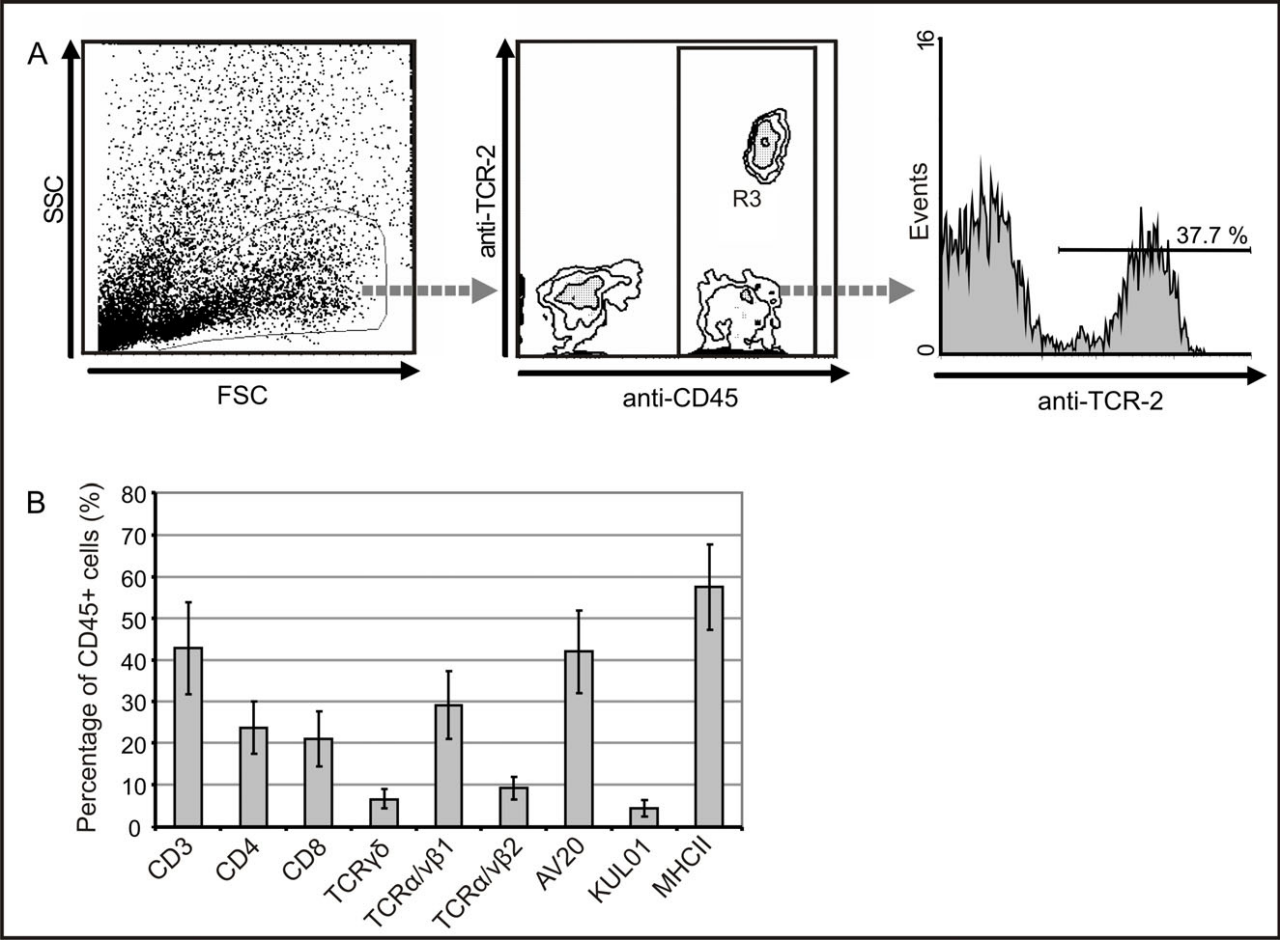
**Leukocytes in spinal ganglia from diseased birds consist of different subpopulations**. For flow cytometric analysis, single cell suspensions from spinal ganglia of diseased birds were stained with a Pan-Leukocyte marker (CD45) and antibodies for T cells (CD3), cytotoxic T cells (CD8), T helper cells (CD4), T cell receptor types TCR1, TCR2 and TCR3 (TCRγδ, TCRα/vβ1, TCRα/vβ2), B cells (AV20), macrophages (Kul01) and MHC class II (2G11). (A) Gating was first performed on cells with leukocyte scatter characteristics, secondly on CD45+ cells before the proportion of different lymphocyte subpopulations of all CD45+ leukocytes was determined. (B) Data expressed as mean ± SD for different subpopulations for five birds analysed.

Flow cytometry identified large numbers of the isolated cells from spinal nerve roots, including DRG, that were CD45-positive leukocytes (Fig. 11A middle panel). The composition of this leukocyte population basically matched the IHC results. The lymphocyte population consisted of 42% B-cells (AV20^+^) and approximately 43% T-cells. The T-cell population split into cytotoxic T-cells and T-helper cells at a ratio of 1:0.94 or, relating to the T-cell receptor, at a ratio of 1:0.18 for αβ T-cells (TCR2 and TCR3) to γδ T-cells (TCR1; Fig. 11B).

About 4% of the isolated leukocytes were monocytes and macrophages (KUL01^+^) and a majority - more than 57% - of the cells carried MHC class II antigens.

### Fluorescence microscopy

In addition to Schwann cells, further DAPI-stained nuclei were detected under the laminin-stained basal lamina of the inner endoneurial sheath. In agreement with the results of the transmission electron microscopy, they were considered to be invading macrophages. As in the IHC samples, IF revealed positive signals for IgG on the surface of myelin sheaths of multiple large myelinated fibres in spinal nerve roots, including DRG, and distal nerve segments.

### Quantitative RT-PCR

**Figure 12:**
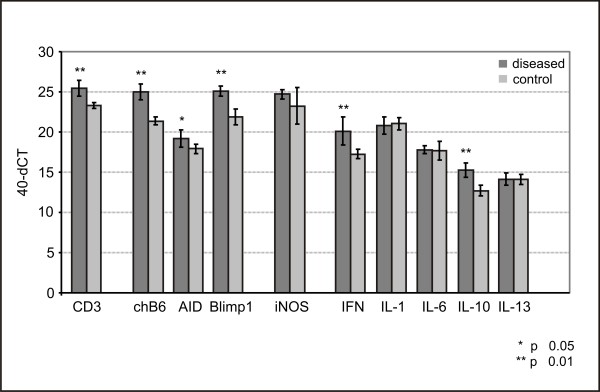
**Relative Gene expression in spinal ganglia of chicken**. The mRNA abundance of candidate genes using quantitative RT-PCR was analysed in ganglia of affected and non-affected chickens of the same age, originated from the same flock. Gene expression, measured using SYBR Green primer assays and Ct values, were normalised against 18S rRNA (= dCT). Data shown as mean ± SD for 40-dCT values for eight animals per group (** p ≤ 0.01; * p ≤ 0.05).

Expression levels of characteristic T- and B-cell genes were significantly increased in spinal ganglia of affected birds in comparison with controls (Figure 12). Averaged differences of 2 and 3.5 CT values (equivalent to a 9-fold and 13-fold increase) were observed for CD3ε and chB6 (a chicken B-cell antigen), respectively, thus confirming the histological and flow cytometric results. In addition to chB6 expression as a marker for immature, mature, and differentiating B-cells in germinal centres (GC), the elevated expression of Blimp-1 and AID with 3 and 1 CTs difference between control and diseased birds indicated that there was also a significant plasma cell activation and induction of hypermutation and class switching events in the analysed spinal ganglia and associated nerve roots.

No differences were observed in the expression levels of the inflammatory cytokines IL-1 and IL-6. Likewise, inducible nitric oxide synthase (iNOS) expression as a marker for activated macrophages showed no significant difference between the two groups. The elevated error bar in the control group originates from a single bird with very low iNOS mRNA levels. Without this animal statistical analysis shows identical expression levels for iNOS in affected and control organs.

Further analysis of cytokine gene expression revealed a significant induction of IFN-γ mRNA in diseased organs (3 CT values corresponding to a 7-fold increases) while the Th2 cytokine IL13 was unchanged. The regulatory cytokine IL-10 was significantly upregulated in ganglia of affected birds (3 CT values, corresponding to 7-fold increase).

### Genotyping

**Figure 13:**
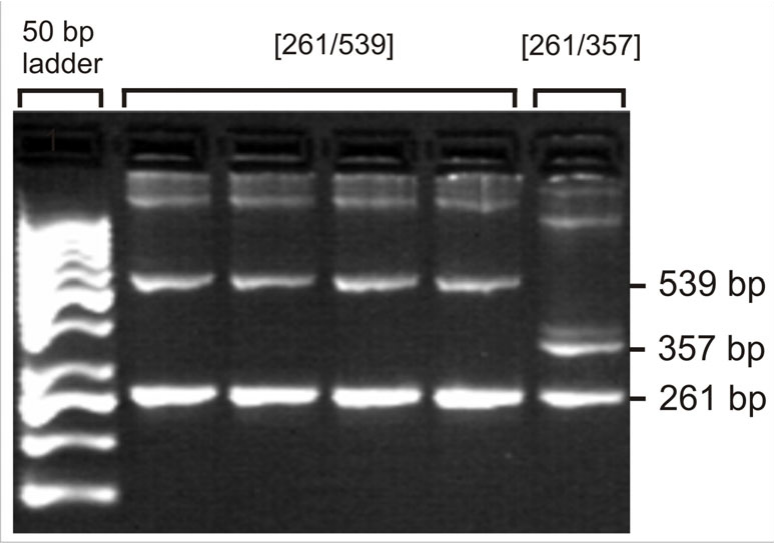
**Fragment length polymorphism of LEI0258**. Blood group genotyping revealed an association between AvIDP and MHC haplotype with a fragment size of 539 bp. The individual with genotype [261/357] was healthy.

The three alleles at locus LEI0258 found in the chicken flock under study corresponded to allele sizes of 261 bp, 357 bp, and 539 bp, respectively (Figure 13). According to this the following genotypes were observed: Genotype *[261/261]*; Genotype *[261/539]*; Genotype *[357/539]*; Genotype *[261/357]*. The frequency of these different genotypes in association with healthy and clinically diseased animals is presented in Table 3. Genotypes *[261/539] *and *[261/357] *occurred in significantly higher numbers of observation (150, 85) than genotypes *[261/261] *and *[357/539] *(18, 12). There was a marked difference between the genotypes in the occurrence of AvIDP symptoms. While the percentage of sick animals was 16.7% and 9.4% in genotypes *[261/261] *and *[261/357]*, this proportion was significantly higher in genotypes *[261/539] *and *[357/539]*, with frequencies of 64.7% and 41.7%, respectively.

**Table 3:** Allele frequency in healthy and clinically AvIDP-diseased animals

	Genotype			
	[261/261]	[261/539]	[357/539]	[261/357]

Healthy	15 (83.3)	53 (35.3)	7 (58.3)	77 (90.6)

Diseased	3 (16.7)	97 (64.7)	5 (41.7)	8 (9.4)

Total	18	150	12	85

Results of a likelihood ratio test revealed a highly significant effect (p < 0.001) of explanatory variables. The factor 'Genotype' is the only one that can be significantly substantiated (p < 0.0001).

Animals of the *[261/539] *genotype had a 9.15-fold higher risk of AvIDP compared to genotype *[261/261] *(CI 2.50-33.04). Compared to genotype *[261/357]*, the risk of showing AvIDP symptoms was even higher for genotype *[261/539] *(17.6-fold, CI 7.9-39.7). Furthermore, the likelihood of genotype *[357/539] *to be affected by AvIDP was higher than those of genotype *[261/357] *by a factor of 6.8. There were neither significant difference between animals of the genotype *[357/539] *and *[261/539]*, nor between animals of the genotypes *[261/357] *and *[261/261] *(Table 4).

**Table 4:** Odds Ratios in pair-wise comparisons for association between genotype and diseases incidence.

Genotype	Odds Ratio and 95% CI*	χ^2^	p
[261/261] vs. **[261/539]**	9.15 (2.50-33.04)	11.4	0.0007

[261/261] vs. [357/539]	3.57 (0.65-19.34)	2.18	0.1397

[357/539] vs. [261/539]	2.56 (0.77-8.46)	2.37	0.1229

[261/357] vs. [261/261]	1.92 (0.45-8.10)	0.79	0.3719

[261/357] vs. **[261/539]**	17.61 (7.90-39.25)	49.2	<0.0001

[261/357] vs. **[357/539]**	6.87 (1.76-26.76)	7.72	0.0054

*CI - Wald confidence interval

### PCR analysis for MDV strain discrimination

PCR analysis of the various organ samples revealed all spleens and some nerve tissues of the vaccinated chickens to be positive for MDV-DNA. However, none of the organ samples scored positive for MDV field strain-DNA in the discriminating PCR. As a proof of principle, all viral DNAs used as controls scored positive for MDV-UL49, but only those from the non-vaccine virus strains RB-1B and BC-1 allowed specific binding of the discrimination primer (UL49disc_fw) and amplification of detectable PCR-products.

## Discussion

During the last decade, an acute paretic syndrome of unclear origin was increasingly reported from flocks of White Leghorn chickens worldwide [16]. In an effort to elucidate the underlying cause and pathobiology of this syndrome we performed an extensive pathohistological and immunological investigation.

All birds of our collective that presented with limb paresis showed severe demyelination of peripheral nerves, with a predilection for craniospinal nerve roots and associated ganglia. This demyelination was associated with multifocal endoneurial infiltration by lymphocytes, plasma cells and macrophages. TEM provided evidence of macrophages invading myelin spirals at the outer mesaxon, between the paranodal loops and Schmidt-Lanterman incisures, leading to stripping off of myelin lamellae from morphologically intact axons. This picture closely resembles human AIDP, which leads us to denote this disease as avian inflammatory demyelinating polyradiculoneuritis (AvIDP) [46,47].

Several pathways have been proposed by which the attention of macrophages is directed towards the myelin sheath. Firstly, autoreactive T-helper cells may secret chemokines attracting macrophages to the endoneurium and, subsequently, activate them via macrophage-activating factors. Activated macrophages also are capable of recognising the Fc-region of auto-antibodies that opsonise myelin epitopes and/or activate the complement system [48]. In AvIDP there is a considerable body of evidence for employment of both pathways. Immunohistological, flow cytometrical and gene expression studies confirm the presence of significant numbers of αβ T-cells of both T-helper and cytotoxic T-cell (CTL) phenotypes. Gene expression profiling further indicated a dominance of a Th1-like immune response as demonstrated by a significant upregulation of IFNγ mRNA and no changes in IL-13 mRNA levels in affected nerves in comparison to healthy controls [49,50]. Th1-like immune responses have been described as taking part in acute EAN and in the initial stages of AIDP; findings which further underline the similarity between AIDP and AvIDP [51,52].

On the other hand, one may ask if the high density of endoneurial plasma cells and deposition of myelin-bound IgG lend credence to a simultaneous recruitment of humoral effector mechanisms [53]. IL-10 expression suggests a Th-cell-dependency rather than a primary humoral response [54]. Morphological examination suggests, and quantitative PCR confirms, that humoral pathways seem to predominate at the stage of AvIDP disease investigated in this study. B-cell and plasma cell activation is reflected by high mRNA expression levels of chB6 (Bu-1) and B lymphocyte-induced maturation protein-1 (Blimp-1) in the affected spinal ganglia [38,55]. Interestingly, IL-13 was - despite the indications for a predominantly humoral pathway - not significantly elevated [56,57], which may be explained by the stage of disease (see below).

The increase in mRNA expression of IL-10 is suggestive of down-regulation of the immune response [54,58,59] and it is in line with the observation of a remission of the clinical deficits soon after the paralytic phase in an additional group of animals subjected to long-term trials (unpublished data; see below).

Furthermore, there was no elevation of IL-1 and IL-6, which contrasts with common observations of an acute inflammatory reaction [60,61]. Even though there is an increase in IFNγ-level, it seems not to have led to the transformation of macrophages into inflammatory macrophages that would have produced higher levels of iNOS in addition to IL-1 and IL-6 [62,63].

In accord with recent data on AIDP and on rodent GBS models [9], it may be concluded that the paraparetic birds in this study were just about to leave a Th1-driven initial stage for a Th2-dominated plateau or even recovery phase. It is very likely that the occurrence of neurological deficits coincides with late a Th1-stage or a Th1-to-Th2-transition, which implies the existence of a considerable preclinical period in disease progression. Because of the spontaneous appearance of disease, and the low incidence (1 per 100 animals) at earlier stages of AvIDP would be very difficult to assess through random sampling in preparetic chickens.

Concerning the natural history of the disease, however, we have launched preliminary longitudinal trials in order to clarify whether AvIDP resembles an acute monophasic disease or a chronic progressive, stagnant or even remitting-relapsing disease with sudden onset. Our preliminary observations indicate that remission and relapse of clinical signs is possible.

In GBS and CIDP, several auto-antibodies have been demonstrated to react with myelin proteins and peripheral nerve gangliosides. Auto-antibodies against myelin proteins P0, P2 and PMP-22 [64] are associated with AIDP and CIDP in humans. These proteins are uniformly distributed throughout the PNS and do not explain either the proximodistal gradient of inflammatory demyelination or the variations in degree of involvement amongst different nerves. Ganglioside composition, however, does vary among different cranial nerves [65]. It therefore has been shown in AIDP and CIDP in humans, and in corresponding animal models in laboratory animals, that the profile of anti-ganglioside antibodies may predict the clinical phenotype [66,67]. Hence, oculomotor nerve involvement is a characteristic feature of the Miller-Fisher syndrome and of GBS with ophthalmoplegia associated with antibodies directed against the ganglioside GQ1b that is particularly abundant in CNIII [65,68]. The binding partners for IgG in AvIDP remain to be identified but the remarkable involvement of CNIII and CNV render similarly distributed avian gangliosides likely target molecules for immunoaggression, even though Miller-Fisher syndrome and GBS with ophthalmoplegia are axonal diseases [48,69].

In analogy to EAN models, the main fraction of recruited T-cells carried αβ T-cell receptors [70]. Even though a contribution of γδ T-cells to human IDP has been documented, indicating a role of non-peptide antigens as triggers for autoaggression [71,72], the prevalence of at least 10% γδ T-cells in AvIDP has to be interpreted with caution since this T-cell population represents about 20 to 50% of the circulating T-cell pool in chickens under physiological conditions [73]. In contrast, humans γδ T-cells represent approximately 1 to 15% of peripheral blood lymphocytes [74].

So far, the aetiology of AvIDP is still undetermined. Preceding events associated with the onset of GBS range from viral, mycoplasmal and bacterial infections to surgery, vaccination, fever treatment and other stressful conditions [75,76]. All chickens, affected and unaffected, originated from one single flock and were raised in an identical environment regarding diet, housing and exposure to environmental pathogens. In contrast to SPF-animals, these chickens are exposed to permanent infection pressures that remain to be controlled by tight polyvaccination management.

Thus, an association with preceding infection, as well as with vaccination, may be involved in disease development via molecular mimicry as has been documented in GBS [76,77].

Outbreaks of viral, mycoplasmal and bacterial infections in chicken flocks are generally limited by strict vaccination programs and by routine health monitoring. However, worldwide distribution, paired with neurotropism and some overlapping clinical and histopathological features, render Marek's disease herpesvirus an important candidate amongst avian infectious agents [78]. Previous studies already have emphasised the similarity of demyelination in Marek's disease (MD) and in EAN [79-81]. Moreover, GBS has been associated with a panel of human herpesviruses, namely Varicella-zoster virus [82-84], cytomegalovirus [84-86], Herpes simplex virus [84] and more frequently Epstein Barr virus [84-86]. We therefore employed a PCR protocol that allows for the discrimination of MDV vaccine and field strains. The results of this PCR analysis strongly suggest that infections with virulent MDV strains should not be considered as the cause of AIDP.

In addition to preceding infections, vaccinations have been reported risk factors for GBS and CIDP in humans. Anteceding immunizations with vaccines against influenza, hepatitis, measles, mumps, and rubella and others infrequently have been associated with GBS [77,87-89]. All chickens in this study were vaccinated in the first few weeks post hatching with inactivated or live vaccines against MDV, Newcastle disease virus, infectious bursal disease virus, avian infectious bronchitis virus, Salmonella spp., and coccidiosis. Hence, multivaccination appears a possible immunological trigger. The potential contribution of infections and/or immunizations to disease development is supported by the observation that SPF animals of the same genetic background do not develop AvIDP. Further trials on a population of unvaccinated chickens are mandatory to evaluate the possible role of vaccination programs for AvIDP development.

While external triggers still remain uncertain, we were able to identify a genetic susceptibility factor confined to the avian major histocompatibility complex (MHC), the so called B-complex. The four genotypes studied displayed marked differences in risk of being affected by AvIDP. Most obvious was the increased risk of genotype *[261/539] *compared to others. In addition, genotype *[357/539] *also showed higher risk of AvIDP compared to genotype *[261/357]*, and, to a lesser extend, compared to genotype *[261/261] *as well.

Moreover, the percentage of AvIDP-affected animals was significantly higher for genotypes *[261/539] *and *[357/539] *than for the other two haplotype combinations. Results suggest an association of marker LEI0258, located in the MHC region of the chicken, with the occurrence of AvIDP as earlier indicated by Bacon et al. [16]. Thereby, the allele with a fragment size of 539 bp seems to be linked to an elevated risk of developing this disease.

Likewise, in the highly susceptible Lewis rats that are commonly used in EAN trials, a certain allele of a MHC-linked gene - amongst further, non-MHC regions - is necessary in the MHC or RT1 region to confer EAE susceptibility in the F2 progeny [90]. Furthermore, HLA-DR2 is associated with a higher susceptibility to CIDP in humans [91]. In GBS, a genetic background is also suspected but has not yet been confirmed [92,93].

To date, EAN is the most frequently used animal model for investigation of immunopathological mechanisms in acute inflammatory demyelinating diseases of the PNS [13]. Even though it strongly resembles AIDP histopathologically, there are several disadvantages and dissimilarities to the human disease in terms of CNS involvement - which is very rare in GBS [20,94] - and a monocausal neuritogenic trigger, that poorly reflects the natural disease development [13].

Like AIDP and EAN, AvIDP is characterised by infiltration of nerve roots and peripheral nerves with macrophages and lymphocytes and, most importantly, a cell-mediated demyelination [13,95]. In AvIDP the CNS is not involved at any time, as is described for the vast majority of GBS cases [20,94].

Compared to experimental immunization with mimicked epitopes, the spontaneous disease development of AvIDP is much closer to the field situation and provides an opportunity to investigate aetiological factors through purposed-based exposure and manipulations of the environment.

A drawback of AvIDP as a disease model lays in the difficulty to identify pre- or subclinical animals and thereby the very early stages of immunopathology, before switching from Th1- to Th2-mediated cascades. However, scientific approaches to AvIDP are facilitated by its availability, reproducibility and economic considerations. In a flock of up to 5000 animals, a mean number of 50 to 200 chickens is affected per 18 weeks of the breeding cycle. In addition, the availability of the chicken genome sequence now greatly facilitates genetic and immunological studies and has lead to the availability of numerous molecular tools for detailed studies [96]. This and the possibility to select susceptible and resistant birds make the AvIDP-chicken a valuable model system for further studies of inflammatory demyelinating polyradiculoneuropathies.

## Conclusions

Sporadic paralysis in juvenile White Leghorn chickens is caused by an inflammatory demyelination of cranial and peripheral nerves. Paralytic stages are associated with humoral immune events that resemble the late stage of AIDP in humans. The natural development of AvIDP is an advantage over EAN regarding research on disease causing factors. MHC-related genetic factors are involved in disease susceptibility. Taken together, AvIDP may serve as a valuable model for further investigations on the aetiology and immunobiology of AIDP as well as its therapy.

## Abbreviations

(AIDP): Acute inflammatory demyelinating polyneuropathy; (AvIDP): avian inflammatory demyelinating polyradiculoneuritis; (CNS): central nervous system; (CN): cranial nerve; (CTL): cytotoxic T cell; (DRG): dorsal root ganglion; (CIDP): chronic inflammatory demyelinating polyneuropathy; (EAN): experimental autoimmune neuritis; (GC): germinal centre; (GSB): Guillain-Barré syndrome; (mAb): monoclonal antibody; (iNOS): inducible nitric oxide synthase; (MHC): major histocompatibility complex; (MD): Marek's disease; (MDV): Marek's disease herpesvirus; (PNS): peripheral nervous system; (TCR): T-cell receptor; (Th): T helper cell.

## Competing interests

The authors declare that they have no competing interests.

## Authors' contributions

KM and BK provided the study concept, design and supervision. SB, SK, ST, SCNS, SW, ARS, and H-CP participated in acquisition of data. SB, SK, KM, and H-CP provided analysis and interpretation. SB, SK, KM, and BK participated in drafting of the manuscript. WS and RP provided critical revision for important intellectual content. All authors read and approved the final manuscript.
